# The association between the observed and perceived neighbourhood food environment and household food insecurity in a low-income district in Lima, Peru

**DOI:** 10.1017/jns.2022.88

**Published:** 2022-09-30

**Authors:** M. Pia Chaparro, Miguel A. Lopez, Julie Hernandez, Jessica D. Brewer, Maria P. Santos, Valerie A. Paz-Soldan

**Affiliations:** 1 Department of Social, Behavioral, and Population Sciences, School of Public Health and Tropical Medicine, Tulane University, 1440 Canal St., suite 2210-16, mail code #8319, New Orleans, LA 70112, USA; 2 Department of International Health and Sustainable Development, School of Public Health and Tropical Medicine, Tulane University, 1440 Canal St., suite 2210, New Orleans, LA 70112, USA; 3 Department of Epidemiology, School of Public Health and Tropical Medicine, Tulane University, 1440 Canal St., suite 2000, New Orleans, LA 70112, USA; 4 Asociación Benéfica PRISMA, Av. Santo Toribio 115, 5to piso, San Isidro, Lima, Peru

**Keywords:** Food access, Food environment, Food insecurity, GIS, Peru

## Abstract

The objective of the present study was to assess the association between the observed and perceived food environment and food insecurity among households with children <18 years in Lima, Peru. This was a cross-sectional study including an income-stratified random sample of households (*n* 329) in Villa el Salvador, a low-income district in Lima, Peru. Data were collected with a household questionnaire – including the Household Food Insecurity Access Scale (HFIAS) and the University of Pennsylvania's Perceived Nutrition Environment Survey (NEMS-P) – and a neighbourhood food outlet census, including recording of food outlets’ GPS coordinates. Three-quarters of the households interviewed were food insecure. Compared with food secure households and adjusting for socio-demographic covariates, food insecure households were more likely to disagree to having easy access (OR 5⋅4; 95 % CI 2⋅1, 13⋅4), high quality (OR 3⋅1; 95 % CI 1⋅7, 5⋅5) and variety (OR 2⋅5; 95 % CI 1⋅4, 4⋅6) of fresh fruits and vegetables in their neighbourhood. About 60 % (513 out of 861) of the food outlets identified in participants’ neighbourhoods were classified as fresh, including markets, bodegas, and fruit and vegetable vendors. There was no difference in distance to fresh food outlets by household food insecurity; all households were on average within 52–62 m from a fresh food outlet (~2-min walk). Despite negative perceptions of their neighbourhood food environment, food insecure households had similar physical access to fresh food sources than their food secure counterparts. Thus, changes to the food environment may not alleviate food insecurity in urban poor areas of Peru.

## Introduction

Food insecurity occurs when a person or a household lacks regular access to sufficient, safe and nutritious foods^(1)^. In 2014–16, the Food and Agriculture Organization (FAO) estimated that 37 % of households in Peru suffered from moderate or severe food insecurity^(2)^. This prevalence jumped to 48 % in 2018–20, however, primarily due to the severe economic downturn experienced as consequence of the COVID-19 pandemic^(2)^. In 2022, food insecurity has continued to increase in Peru, now reaching a prevalence of 50⋅3 %, the highest in South America, which translates into 16 million Peruvians struggling to put food on their table^(3)^.

Living in poverty is a key determinant of food insecurity in Peru, as it is across the Latin American region^(4,5)^. However, additional information on food insecurity determinants, particularly those amenable for intervention, is lacking in this setting. In particular, the potential role that the neighbourhood food environment may play in influencing food insecurity risk is unknown. Neighbourhoods are an attractive venue for population-level interventions, with the capacity to influence individual-level behaviours at a larger scale and, thus, being more cost-effective^(6)^. Previous studies in high-income settings have reported that characteristics of the food environment, including presence of and distance to food outlets, are associated with perceived food quality and food access^(7,8)^, the latter, in particular, being linked to food security.

The goal of the present study was to assess the association between the observed and perceived food environment and food insecurity among households with children in Villa El Salvador, one of the most populous districts in Lima, Peru. Our objectives were to investigate whether (1) food insecurity was associated with perceptions of access, quality and cost of food in participants’ neighbourhoods and their most visited food outlet; and (2) food (in)security varied based on proximity to fresh food outlets.

## Experimental methods

### Study setting

This study took place in Villa El Salvador, the fifth largest district in Lima, the capital of Peru. Villa El Salvador started as a squatter settlement of internal migrants in the 1970s, with residents shaping the development of the district from the ground-up^(9)^. Over time, Villa El Salvador residents demanded and obtained government services, including running water, electricity and sewage, and formal ownership of the land^(9)^. Moreover, the Villa El Salvador community was actively involved in social movements that led to the establishment of some of the largest government food assistance programs in Peru, such as *El Vaso de Leche* (Glass of Milk program) and *Comedores Populares* (Community Kitchens)^(10)^. While still considered a low-income district in comparison to the rest of the city, Villa el Salvador is now more socioeconomically diverse^(11)^, with middle-income residential areas intermixed with businesses, and low income, new settlement areas still lacking basic services.

### Study design

Data for this cross-sectional study, including a household questionnaire and a census of neighbourhood food outlets, were collected between June and August 2019 in Villa El Salvador. The household questionnaire was piloted in two phases with fourteen and then five conveniently sampled households to validate language and understanding of the questions. Data were collected from a random selection of 450 city blocks, 150 from each of three income strata (low income, lower middle income, and middle income) determined by average per capita household income, according to the Peruvian National Institute of Statistics and Informatics^(11)^. One household per city block was selected via systematic sampling. The house in the northeast corner of the city block was approached first; if a participant in that household could not be interviewed because of ineligibility, refusal, or not answering, we attempted contact in the house neighbouring to the right, and then the house neighbouring to the left, continuing with every house on the left until a questionnaire was completed or all the houses in that city block were attempted. Inclusion criteria to participate in the study included: the participant had to be at least 18 years old, in charge of purchasing food for the household, and living in a household with at least one minor (<18 years old). A total of 2267 interviews were attempted, but 776 (34⋅2 %) households did not answer the door; from the remaining 1491 households that answered the door, 385 (25⋅8 %) refused to participate and 777 (52 %) were not eligible. Therefore, 329 interviews were completed, which accounts for 46 % of the eligible households who answered the door. There were no significant differences in neighbourhood income strata between households not answering the door and those answering the door (ANOVA; *P*-value = 0⋅18), nor between refusals and participants (ANOVA; *P*-value = 0⋅09). All responses to the household questionnaire were gathered in Spanish by trained staff from Lima, Peru using REDCap on a tablet. Each questionnaire took approximately 30 min to complete, and the participant was provided with a fruit basket at the conclusion of the survey to compensate them for their time.

The questionnaire included questions on demographic and socioeconomic characteristics, household food security, food assistance program participation, informal methods of food access, as well as perceptions of the access, quantity, and quality of foods in the participants’ neighbourhood, and perceptions of access, quality, and cost of foods in frequently visited food outlets. For questions related to the participants’ neighbourhood food environment, the participant was asked to think of their neighbourhood as the area accessible within 20 min of walking from their home. In terms of the most regularly visited food outlets, participants were asked to list their top three most visited stores where they purchase groceries and all questions following were in reference to those; 99 % of the sample reported data on their number one most visited store, with only 88 and 47 % reporting on their second and third most visited store, respectively. Given the extensive amount of data already covered in this paper, we only report results for the most visited food outlet reported by participants.

### Outcome variable

Household food security status was determined using the Household Food Insecurity Access Scale (HFIAS)^(12)^, a scale that has been previously validated for use in Peru^(13)^. The HFIAS includes nine questions asking whether specific experiences associated with food insecurity occurred within the household in the previous 4 weeks, with response options ‘yes’ (1 point) or ‘no’ (0 points). If the respondent answered yes, follow-up questions are asked to measure how often the experience occurred, including ‘rarely’ (1 point), ‘sometimes’ (2 points) or ‘often’ (3 points). Based on the HFIAS score, which ranges from 0 to 27, and following standard procedures^(12)^, households are classified as having food security, mild food insecurity, moderate food insecurity or severe food insecurity. For multivariable analysis, food security status was dichotomised into food secure or food insecure (combining the categories of mild, moderate and severe food insecurity).

### Perceived food environment

Questions related to the perceived food environment of the participant's neighbourhood and their most frequented food outlets were adapted from the University of Pennsylvania's Perceived Nutrition Environment Survey (NEMS-P)^(14)^ and translated into Spanish. Questionnaire items are displayed in Supplementary Table. Statements related to the variety, accessibility, quality, and cost of food products had five Likert-scale response options ranging from ‘completely disagree’ to ‘completely agree’ or four response options ranging from ‘very easy’ to ‘very difficult.’ For analysis, answers were dichotomised to either ‘agree’ (completely agree and agree) or ‘does not agree’ (neither agree nor disagree, disagree and totally disagree), and ‘easy’ (very easy and easy) or ‘not easy’ (difficult and very difficult) due to small sample size in some cells of the original response options.

### Observed food environment

After the household questionnaire was completed and the house GPS coordinates were recorded, a census of the food outlets in the household's city block was carried out. The household's city block was defined as the group of houses or buildings sharing the same ‘square’ as the household (i.e. including all four corners), without crossing any streets. The food outlet census included recording the GPS coordinates of all food outlets as well as the type of food outlet, with the following pre-determined categories obtained from pilot testing in two different Lima districts: market, supermarket, bodega, restaurant, street vendor and other, with subcategories available for bodegas (3 sub-categories), restaurants (7 sub-categories), and street vendors (7 sub-categories). For the present study, we focused on food outlets selling fresh foods that could be eaten raw or prepared at home, which included markets, bodegas, and fruit and vegetable street vendors (there were no supermarkets in the city blocks examined). In other words, the observed food environment was operationalised based on the number of fresh food outlets in the area, and their location in reference to the interviewed households.

### Statistical analyses

Descriptive statistics of study participants and their households were estimated by the presence and severity of food insecurity. Bivariate associations between perceived food environment variables – both in terms of participants’ neighbourhoods and participants’ most frequented food outlet – and household food insecurity were established with chi-square analyses. Multivariable logistic regression models were run to estimate the association between perceived food environment variables (independent outcomes) and food insecurity (predictor), controlling for age of the respondent (in years), gender of the respondent (man *v*. woman), highest education level of the respondent (less than high school graduate, high school graduate, or some college or trade school and beyond), and weekly household food expenditure per person (in Peruvian soles). Other demographic characteristics such as marital status and household occupation were not adjusted for in multivariable analyses because they were not associated with food insecurity in this sample. Analyses were conducted using SAS v9.4 (SAS Institute Inc., Cary, NC, USA) with a *P*-value of <0⋅05 considered statistically significant.

In terms of the observed food environment, we used GPS coordinates to plot the households and food sources datasets in QGIS (Version 3.12 București), then produced four shapefiles corresponding to each category of household food security (food security, mild food insecurity, moderate food insecurity and severe food insecurity). We also produced a shapefile including the selected fresh food outlets in the neighbourhood. All shapefiles were re-projected in WGS84 – UTM Zone 18 S. We conducted a distance matrix analysis to evaluate average distance between households and the nearest fresh food outlet, based on household food security status. For each category of household food security, we ran the *Nearest Hub* function using ‘as the crow flies’ distance to estimate average distance between each household and the nearest fresh food outlet. We then averaged that distance by each food security status category.

### Ethical standards

This study was conducted according to the guidelines laid down in the Declaration of Helsinki and all procedures involving research study participants were approved by the Asociación Benéfica PRISMA's Institutional Committee of Ethics in Research (IRB# CE024220) and the Tulane University Human Research Protection Office (IRB# 2019–614). Written informed consent was obtained from all study participants.

## Results

Table 1 displays the selected characteristics of study participants by the presence and severity of food insecurity. The sample consisted of 329 participants (mean age 40⋅3 years, 92⋅1 % women). About 77 % of households were food insecure, including 14⋅9 % with mild food insecurity, 24⋅0 % with moderate food insecurity and 38⋅3 % with severe food insecurity. Education level was higher among the food secure as was the proportion of self-employed or employed, compared to the food insecure. Weekly household food expenditures per person were also higher among food secure households (43 ± 20 Peruvian soles) compared to food insecure households (34 ± 14 Peruvian soles). Finally, the proportion of households owning a motor vehicle was higher among the food secure (33 %) compared to the food insecure (21 %).

**Table 1.** Selected demographic characteristics of participants by household food security status, Villa el Salvador, Lima, Peru (*n* 329)

		Food insecurity			
Household characteristics	Food security	Mild	Moderate	Severe	Total
No. of households, *n* (%)	75 (22⋅8)	49 (14⋅9)	79 (24⋅0)	126 (38⋅3)	254 (77⋅2)
Age of respondent (years), mean (sd)	39⋅3 (12⋅4)	37⋅0 (11⋅2)	37⋅4 (12⋅3)	44⋅2 (13⋅1)	40⋅6 (12⋅9)
Gender of respondent (female), *n* (%)	64(85⋅3)	45 (91⋅8)	77 (97⋅5)	117 (92⋅9)	239 (94⋅1)
Education of respondent, *n* (%)					
<High school graduate	11 (14⋅7)	7 (14⋅3)	21 (26⋅6)	58 (46⋅0)	86 (33⋅9)
High school graduate	24 (32⋅0)	23 (46⋅9)	28 (35⋅4)	40 (31⋅8)	91 (35⋅8)
Some college or technical school and above	40 (53⋅3)	19 (38⋅8)	30 (38⋅0)	28 (22⋅2)	77 (30⋅3)
Marital status of respondent, *n* (%)					
Never married	8 (10⋅7)	4 (8⋅2)	7 (8⋅9)	18 (14⋅3)	29 (11⋅4)
Living with a partner	39 (52⋅0)	31 (63⋅3)	40 (50⋅6)	58 (46⋅0)	129 (50⋅8)
Married	24 (32⋅0)	11 (22⋅5)	19 (24⋅1)	29 (23⋅0)	59 (23⋅2)
Separated, divorced or widowed	4 (5⋅3)	3 (6⋅1)	13 (16⋅5)	21 (16⋅7)	37 (14⋅6)
Occupation of respondent, *N* (%)					
Not working	44 (58⋅7)	35 (69⋅4)	52 (65⋅8)	84 (66⋅7)	170 (66⋅9)
Self-employed	23 (30⋅7)	10 (20⋅4)	17 (21⋅5)	29 (23⋅0)	56 (22⋅1)
Employed	8 (10⋅7)	5 (10⋅2)	10 (13⋅3)	13 (10⋅3)	29 (11⋅0)
Weekly household food expenditure per person (Peruvian soles), mean (sd)	43⋅0 (20⋅0)	37⋅9 (14⋅1)	36⋅4 (13⋅5)	30⋅8 (14⋅4)	34⋅0 (14⋅3)
Household vehicle ownership, *n* (%)	24 (32⋅9)	13 (26⋅5)	18 (22⋅8)	22 (17⋅5)	53 (20⋅9)

About three-quarters of the sample agreed it is easy to purchase fresh fruits and vegetables in their neighbourhoods, with 48 % of participants agreeing these fresh fruits and vegetables are of high quality and 57 % agreeing there is a large selection of them available (Table 2). In turn, only half of participants agreed it is easy to purchase low-fat products (i.e. low-fat dairy products) in their neighbourhood, 42 % agreed these products are of high quality, and 43 % agreed there is a large selection of them available. These neighbourhood perception variables varied by household food security status, with percent agreement with the accessibility, quality, and variety statements being lower for food insecure households compared to food secure households (Table 2). Table 3 displays adjusted associations between a participant's perception of their neighbourhood food environment and household food security status. Multivariable logistic regression models indicate that, in general, food insecure households were more likely to report negative perceptions of their neighbourhood food environment in terms of accessibility, quality, and selection of fresh fruits and vegetables and low-fat products. For example, after adjusting for age, gender, education, and weekly household food expenditures per person, participants who lived in food insecure households were 5, 3 and 2⋅5 times more likely to state they ‘did not agree’ to having accessible, high quality, and varied fresh fruits and vegetables in their neighbourhood, respectively, compared to individuals in food secure households (Table 3). Similar associations albeit of lesser magnitude were found for low-fat food products.

**Table 2.** Bivariate associations between household food security status and participants’ perceived food environment, including participants’ neighbourhood food environment and their most frequented food establishment, Villa el Salvador, Lima, Peru (*n* 329)

	Total	Food secure	Food insecure	*P*-value
Neighbourhood food environment (% who responded ‘completely agree' or ‘agree’)				
It is easy to purchase fresh fruits and vegetables in my neighbourhood	75⋅5	92⋅0	70⋅6	0⋅0002
The fresh fruits and vegetables in my neighbourhood are of high quality	47⋅7	66⋅7	42⋅1	0⋅0002
There is a large selection of fresh fruits and vegetables in my neighbourhood	56⋅9	70⋅7	52⋅8	0⋅0060
It is easy to purchase low-fat products in my neighbourhood	50⋅5	61⋅3	47⋅2	0⋅0319
The low-fat products in my neighbourhood are of high quality	41⋅9	54⋅7	38⋅1	0⋅0107
There is a large selection of low-fat products in my neighbourhood	42⋅8	52⋅9	40⋅1	0⋅0670
Most frequented food outlet (% who responded ‘completely agree’ or ‘agree’)				
There is a large selection of food in most frequented food outlet	89⋅0	89⋅3	88⋅9	0⋅9140
The food in most frequented food outlet is of high quality	73⋅4	80⋅0	71⋅4	0⋅1403
The price of food in most frequented food outlet is affordable (% who responded ‘very easy’ or ‘easy’)	80⋅1	78⋅7	80⋅6	0⋅7190
How easy it is to find fresh fruits and vegetables in most frequented food outlet	82⋅3	86⋅7	81⋅0	0⋅2554
How easy it is to find low-fat products in most frequented food outlet	73⋅9	73⋅3	74⋅1	0⋅8939
How easy it is to find lean meat in most frequented food outlet	51⋅5	50⋅7	51⋅8	0⋅8640
How easy it is to find sugary drinks or carbonated beverages in most frequented food outlet	92⋅7	88⋅0	94⋅1	0⋅0779
How easy it is to find candy and junk food like potato chips in most frequented food outlet	89⋅0	88⋅0	89⋅3	0⋅7548

**Table 3.** Results from multivariable^a^ logistic regression models predicting accessibility, quality, and variety of fruits and vegetables and low-fat products in participants’ neighbourhoods by household food insecurity, Villa El Salvador, Lima, Peru (*n* 329)

	Food insecurity	
	OR	95 % CI
Accessibility to fresh fruits and vegetables in neighbourhood^b^	5⋅35	2⋅14–13⋅43
Quality of fresh fruits and vegetables in neighbourhood^b^	3⋅08	1⋅72–5⋅54
Variety of fresh fruits and vegetables in neighbourhood^b^	2⋅52	1⋅38–4⋅62
Accessibility of low-fat products in neighbourhood^b^	2⋅21	1⋅25–3⋅92
Quality of low-fat products in neighbourhood^b^	1⋅94	1⋅11–3⋅39
Variety of low-fat products in neighbourhood^b^	1⋅79	1⋅03–3⋅13

a All analyses were adjusted by participants’ age, gender, education level and household weekly food expenditures per person.

b Predicting participant stating that they do not agree (*v*. agree) to having adequate access/quality/variety of fruits and vegetables or low-fat products in their neighbourhood (outcome) by food insecurity (predictor).

As for participants’ most frequented food outlet, 81 % reported this food outlet to be a market, while 13⋅5 % reported this outlet to be a corner store. The majority (56⋅7 %) of participants reported that their most frequently visited food outlet was within a 0–10-min walk from home, with 23⋅6 % reporting the outlet to be within a 11–20-min walk from home. Over 70 % of participants (73⋅2 %) reported walking to their most frequented food outlet; 13⋅9 % reported using a bus or other public transportation, and 10⋅8 % a personal motor vehicle (car or motorcycle). Neither type of most frequently visited outlet nor mode of transportation to reach this food outlet varied by household food security status. However, food secure households reported being closer to their most visited food store (70⋅7 % within a 0–10-min walk) compared to food insecure households (52⋅4 % within a 0–10-min walk; chi-square *P*-value = 0⋅0394).

Overall, participants had positive perceptions of their most frequented food outlet, with most of them agreeing their most frequented food outlet had a large selection of food, which is of high quality and affordable; these perceptions did not vary by household food security status (Table 2). Similarly, most participants stated it is easy to find both healthy (fresh fruits and vegetables, low-fat products and lean meat) and less healthy (sugary drinks, candy, chips) foods in their most frequented store, with no variations by household food security status. Table 4 displays adjusted associations between participants’ perceptions of their most frequented food outlet and household food security status. Adjusting for age, gender, education and weekly household food expenditures, only quality of food in most frequented food outlet and ease of finding sugary drinks in most frequented food outlet were significantly associated with household food security. Food insecure households were twice as likely to state they ‘did not agree’ that the food in their most frequented food outlet is of high quality, compared to food secure households. On the other hand, food insecure households were less than half as likely to report that ‘it is not easy’ to find sugary drinks in their most frequented food outlet, compared to their food secure counterparts (Table 4).

**Table 4.** Results from multivariable^a^ logistic regression models predicting variety, quality, and cost of foods as well as accessibility of different food items in participants’ most frequented food outlet by household food insecurity, Villa El Salvador, Lima, Peru (*n* 329)

	Food insecurity	
	OR	95 % CI
Variety of food in most frequented food outlet^b^	1⋅22	0⋅54–2⋅96
Quality of food in most frequented food outlet^b^	2⋅27	1⋅12–4⋅59
Price of food in most frequented food outlet^b^	0⋅92	0⋅47–1⋅80
Ease of finding fresh fruits and vegetables in most frequented food outlet^c^	1⋅24	0⋅57–2⋅70
Ease of finding low-fat products in most frequented food outlet^c^	1⋅23	0⋅65–2⋅33
Ease of finding lean meat in most frequented food outlet^c^	1⋅08	0⋅62–1⋅88
Ease of finding sugary drinks in most frequented food outlet^c^	0⋅37	0⋅14–0⋅94
Ease of finding junk food in most frequented food outlet^c^	0⋅73	0⋅31–1⋅71

a All analyses were adjusted by participants’ age, gender, education level and household weekly food expenditures per person.

b Predicting participant stating that they do not agree (*v*. agree) to having adequate variety/quality/price of food in their most frequented food outlet (outcome) by food insecurity (predictor).

c Predicting participant stating it is not easy (*v*. easy) finding each type of food in their most frequented food outlet (outcome) by food insecurity (predictor).

**Fig. 1.**
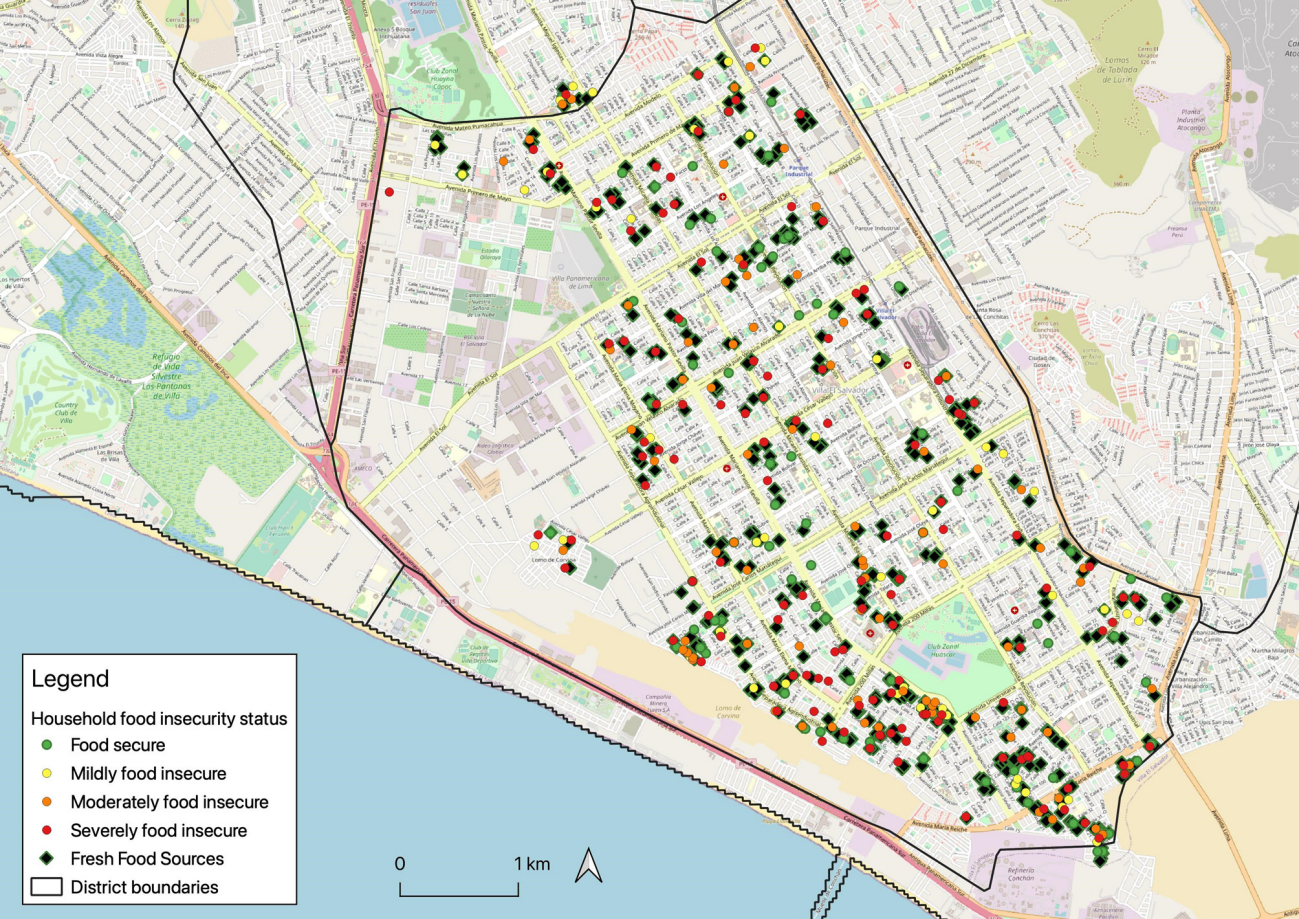
Map of surveyed households (*N* = 329) by household food security status, and fresh food outlets present in the city blocks of surveyed households (*N* = 513), Villa El Salvador, Lima, Peru.

As for the observed food environment, the food outlet census identified 861 food outlets in the 329 city blocks assessed; 513 of these were fresh food outlets and, thus, included in the analysis. There were no supermarkets in the 329 city blocks assessed. Table 5 summarises the distance between surveyed households (*N* = 325, 4 records were dropped because of incomplete GPS coordinates) by household food security status and the nearest fresh food outlet (markets, bodegas, and fruit and vegetable street vendors), while Fig. 1 visually displays the location of the households in reference to fresh food outlets. There were virtually no differences between the distance that food secure households (61⋅9 m) and all categories of food insecure households (52–54 m; Table 5) had to cover to reach one of these fresh food sources. In fact, households with greater food insecurity tended to be located closer to a fresh food source, and all distances were overall very short, equivalent to a ~2 min walk.

**Table 5.** Distance to the nearest fresh food source by household food security status, Villa El Salvador, Lima, Peru (*n*  325)

Food security status	N	Mean distance (metres)	Standard deviation	Minimum distance	Maximum distance
Food security	81	61⋅85	57⋅56	0⋅00	274⋅34
Mild food insecurity	48	54⋅30	48⋅79	0⋅00	222⋅39
Moderate food insecurity	76	53⋅48	52⋅24	0⋅00	268⋅19
Severe food insecurity	120	52⋅14	62⋅12	0⋅00	532⋅65
**Total**	**325**	**55⋅19**	**56⋅81**	**0⋅00**	**532⋅65**

## Discussion

The present study sought to assess the association between the observed and perceived neighbourhood food environments and food insecurity among households with children in a district in Lima, Peru. In terms of the perceived food environment, we evaluated participants’ perceptions of their neighbourhood food environment as well as of their most often visited store. Overall, compared to food secure households, food insecure households had worse perceptions of their neighbourhood food environment in terms of accessibility, quality, and selection of fresh produce and low-fat products. Moreover, perceptions of the most frequented food outlet were worse for food insecure households, compared to the food secure, in terms of food quality. As for the observed food environment, we found that ~60 % of all food outlets available in the study area were considered fresh food outlets and that all households, regardless of food security status, lived within a ~2-min walk to these outlets.

While physical accessibility to fresh food sources was not an issue in participants’ neighbourhoods based on observed data, food insecure households were five times more likely to respond in the negative to the statement ‘It is easy to purchase fresh fruits and vegetables in my neighborhood,’ compared to food secure households. It is possible that food insecure respondents interpreted this question in terms of physical *and* monetary access, which could explain the apparent contradictory findings. Participants’ positive views of their most frequented food outlets lends support to this hypothesis too, as the majority of participants reported reaching their most frequented food outlet via a short walk, highlighting their ease in physically accessing stores. Several studies conducted in the United States have reported poor-to-moderate correlations between observed and perceived measures of the neighbourhood food environment^(15–17)^. A study focused on households with children in eight counties in South Carolina, USA, found that households with very low food security (the equivalent to *severe food insecurity* in this study) reported poorer perceptions of availability, quality, and affordability of fruits and vegetables and availability of low-fat products, compared to food secure households, even though there were no differences between the groups in distance to the nearest supermarket.^(8)^ On the other hand, a study focused on Hispanics living in New York City found that proximity to fruit and vegetable vendors was associated with increased perceptions of fresh fruits and vegetables availability.^(7)^ Unfortunately, comparable studies in Latin America are lacking^(18)^, and there is enough evidence to suggest that findings related to the impact of food environments to nutritional outcomes in high-income countries are not directly applicable to low- and middle-income countries, given the vast diversity of local food systems across the world^(19)^.

Our findings indicate that recommendations to increase physical access to fresh foods might not be sufficient to ameliorate food insecurity as fresh food sources abound in the studied area. A recent report from the Committee of World Food Security suggests increasing physical access to healthy foods as key to improving nutrition in low- and middle-income countries^(20)^. However, a previous report on food insecurity interventions specific to Latin America suggests that food availability is not much of an issue in the region, with monetary access and food utilisation being more important^(21)^. This is consistent with other research, highlighting individual- and family-level factors like income, education, and household composition as the most important determinants of food insecurity in Latin American countries^(4,5)^. In a mixed-methods study conducted in three regions of Peru, Vargas and Penny^(22)^ found that food insecure participants from Lima and those living in the urban areas of the Amazon basin discussed living day-to-day, with little to no food stocks. Thus, proximity to fresh food sources may be irrelevant for food insecure households when economic constraints are so severe.

The strengths of the present study include the use of a stratified random sample of city blocks for data collection, with the inclusion of low income, lower middle income, and middle income areas; objective measures to identify the location of households and nearby fresh food outlets by the use of GPS coordinates; and validated tools to measure food insecurity^(11,13)^ and the perceived food environment^(14)^. Limitations include our focus on just one district in Lima, limiting the generalizability of our findings. In addition, even though items from NEMS-P included in this study have been previously validated^(14)^ and pre-tested for comprehensibility in Spanish for the purpose of this study, validity of this tool in Latin American settings has not been confirmed. In terms of the observed food environment analysis, the *Nearest Hub* function used uses ‘as the crow flies’ distance rather than calculations based on street networks. In urban areas with a dense street network, however, this is a reasonable approximation. Finally, even though our observed food environment analysis included different types of food outlets – including different price points – we did not collect data on actual food prices, limiting our comparisons with the perceived food environment findings in this regard.

In conclusion, the present study found that food insecure households had worse perceptions of their neighbourhood food environment in terms of accessibility, quality, and selection of fruits and vegetables and low-fat products, compared to food secure households, even though proximity to fresh food outlets was not associated with food insecurity. Among urban poor city dwellers in Peru, changes to the physical neighbourhood environment may not alleviate food insecurity. Given the continued increase in food insecurity in Peru in light of the economic downturn and food supply issues triggered by COVID-19 and exacerbated by the Russian-Ukrainian war^(2,3)^, identifying successful interventions to make food more affordable for all Peruvians is warranted.

## Supplementary material

For supplementary material accompanying this paper visit https://doi.org/10.1017/jns.2022.88. click here to view supplementary material

## References

[1] Food and Agriculture Organization (FAO) Food Security Programme (2008) An Introduction to the Basic Concepts of Food Security. Available from: http://www.fao.org/3/a-al936e.pdf (accessed 1 October 2021).

[2] Food and Agriculture Organization (FAO), International Fund for Agricultural Development (IFAD), UNICEF, World Food Programme (WFP), World Health Organization (2021) The State of Food Security and Nutrition in the World. Transforming Food Systems and Food Security, Improved Nutrition and Affordable Healthy Diets for All. Available from: http://www.fao.org/3/cb4474en/cb4474en.pdf (accessed 1 October 2021).

[3] Food and Agriculture Organization (FAO), International Fund for Agricultural Development (IFAD), UNICEF, World Food Programme (WFP), World Health Organization (2022) The State of Food Security and Nutrition in the World. Repurposing Food and Agricultural Policies to make Healthy Diets more Affordable. Available from: https://www.fao.org/3/cc0639en/cc0639en.pdf (accessed 30 August 2022).

[4] Smith MD, Kassa W & Winters P (2017) Assessing food insecurity in Latin American and the Caribbean using FAO's food insecurity access scale. Food Policy 71, 48–61.

[5] Rezende Machado de Sousa L, Saint-Ville A, Samayoa-Figueroa L, (2019) Changes in food security in Latin America from 2014 to 2017. Food Secur 11, 503–513.

[6] Rose G (2001) Sick individuals and sick populations. Int J Epidemiol 30, 427–432. 11416056 10.1093/ije/30.3.427

[7] Co MC & Bakken S (2018) Influence of the local food environment on Hispanics’ perceptions of healthy food access in New York city. Hisp Health Care Int 16, 76–84. 30081666 10.1177/1540415318788068 PMC6117217

[8] Ma X, Liese AD, Bell BA, (2016) Perceived and geographic food access and food security status among households with children. Public Health Nutr 19, 2781–2788. 27133939 10.1017/S1368980016000859 PMC5588026

[9] Bartesaghi-Koc C (2014) Squatter settlements as social catalysts towards a sustainable urban development: a positive look at the case of Villa El Salvador, Lima-Peru. *11th Symposium of International Urban Planning and Environment Association*. La Plata, Argentina.

[10] Copestake J (2008) Multiple dimensions of social assistance: the case of Peru's ‘glass of milk’ programme. J Develop Stud 44, 545–561.

[11] Instituto Nacional de Estadística e Informática (INEI) (2016) *[*Stratified Maps of Metropolitan Lima at the Neighborhood-Block Level Based on per Capita Household Income 2016*] (in Spanish)*. Available from: https://www.inei.gob.pe/media/MenuRecursivo/publicaciones_digitales/Est/Lib1403/index.html (accessed 1 October 2021).

[12] Coates J, Swindale A & Bilinsky P (2007) Household Food Insecurity Access Scale (HFIAS) for Measurement of Food Access: Indicator Guide, Version 3. Food and Nutrition Technical Assistance (FANTA) III and USAID. Available from: https://www.fantaproject.org/sites/default/files/resources/HFIAS_ENG_v3_Aug07.pdf (accessed 1 October 2021).

[13] Psaki S, Bhutta ZA, Ahmed T, (2012) Household food access and child malnutrition: results from the eight-country MAL-ED study. Popul Health Metr 10, 24. 23237098 10.1186/1478-7954-10-24 PMC3584951

[14] Green SH & Glanz K (2015) Development of the perceived nutrition environment measures survey. Am J Prev Med 49, 50–61. 26094227 10.1016/j.amepre.2015.02.004

[15] Barnes TL, Bell BA, Freedman DA, (2015) Do people really know what food retailers exist in their neighborhood? Examining GIS-based and perceived presence of retail food outlets in an eight-county region of South Carolina. Spat Spatiotemporal Epidemiol 13, 31–40. 26046635 10.1016/j.sste.2015.04.004 PMC4457938

[16] Gustafson AA, Sharkey J, Samuel-Hodge C, (2011) Perceived and objective measures of the food store environment and the association with weight and diet among low-income women in North Carolina. Public Health Nutr 14, 1032–1038. 21324229 10.1017/S1368980011000115

[17] Lucan SC, Hillier A, Schechter CB, (2014) Objective and self-reported factors associated with food environment perceptions and fruit-and-vegetable consumption: a multilevel analysis. Prev Chronic Dis 11, E47. 24674635 10.5888/pcd11.130324 PMC3970773

[18] Turner C, Kalamatianou S, Drewnoski A, (2020) Food environment research in low- and middle-income countries: a systematic scoping review. Adv Nutr 11, 387–397. 31079142 10.1093/advances/nmz031 PMC7442349

[19] Turner C, Aggarwal A, Walls H, (2018) Concepts and critical perspectives for food environment research: a global framework with implications for action in low- and middle-income countries. Glob Food Sec 18, 93–101.

[20] HLPE (2017) Nutrition and Food Systems. A Report by the High Level Panel of Experts on Food Security and Nutrition of the Committee on World Food Security, Rome. Available at: http://www.fao.org/3/a-i7846e.pdf (accessed 1 October 2021).

[21] Rose D (2008) Interventions to reduce household food insecurity: a synthesis of current concepts and approaches for Latin America. Rev Nut 21S, 159s–173s.

[22] Vargas S & Penny ME (2009) Measuring food insecurity and hunger in Peru: a qualitative and quantitative analysis of an adapted version of the USD's food insecurity and hunger module. Public Health Nutr 13, 1488–1497. 19968898 10.1017/S136898000999214X

