# Supplementary material for: The association between the observed and perceived neighbourhood food environment and household food insecurity in a low-income district in Lima, Peru
Source: J Nutr Sci. 2022 Sep 30;11:e86. doi: 10.1017/jns.2022.88 (PMC9554428; doi:10.1017/jns.2022.88)
Supplement: Supplementary file 1 [file S204867902200088Xsup001.docx]

**Supplementary Table:** Included questionnaire items related to the perceived food environment*

| **Composite Item** | **Survey Item** | **Response options** |
| --- | --- | --- |
| **Neighborhood food environment** |  |  |
| Accessibility of fresh fruits and vegetables in neighborhood | It is easy to purchase fresh fruits and vegetables in my neighborhood. | 1-5^†^ |
| Quality of fresh fruits and vegetables in neighborhood | The fresh fruits and vegetables in my neighborhood are of high quality. | 1-5^†^ |
| Variety of fresh fruits and vegetables in neighborhood | There is a large selection of fresh fruits and vegetables in my neighborhood. | 1-5^†^ |
| Accessibility of low-fat products in neighborhood | It is easy to purchase low-fat products such as low-fat milk and lean meat in my neighborhood. | 1-5^†^ |
| Quality of low-fat products in neighborhood | The low-fat products in my neighborhood are of high quality. | 1-5^†^ |
| Variety of low-fat products in neighborhood | There is a large selection of low-fat products such as low-fat milk and lean meat in my neighborhood. | 1-5^†^ |
| **Most frequented food outlets** | |  |
| Distance to food outlet(s) | [Name of food outlet] is near my house. | 1-5^†^ |
| Variety of food in food outlet(s) | There is a large selection of food in [name of food outlet]. | 1-5^†^ |
| Quality of food in food outlet(s) | The food in [name of food outlet] are of high quality. | 1-5^†^ |
| Cost of food in food outlet(s) | The price of food at [name of food outlet] is affordable. | 1-5^†^ |
| Availability of fresh fruits and vegetables in food outlet(s) | How easy it is to find fresh fruits and vegetables at [name of food outlet]. | 1-4^‡^ |
| Availability of low-fat dairy products in food outlet(s) | How easy it is to find low-fat products, such as low-fat milk and low-fat yogurt at [name of food outlet]. | 1-4^‡^ |
| Availability of lean meat in food outlet(s) | How easy it is to find lean meat in [name of food outlet]. | 1-4^‡^ |
| Availability of sugary drinks or carbonated beverages  in food outlet(s) | How easy it is to find sugary drinks or carbonated beverages in [name of food outlet]. | 1-4^‡^ |
| Accessibility to junk food in food outlet(s) | How easy it is to find candy and junk food like potato chips in [name of food outlet]. | 1-4^‡^ |

^*^Adapted from the University of Pennsylvania’s Nutrition Environment Measures Survey (NEMS-P)^(12)^

^†^Response options: 1=Completely agree, 2=agree, 3=neither agree nor disagree, 4=disagree, 5=completely disagree.

^‡^Response options: 1=Very difficult, 2=difficult, 3=easy, 4=very easy.
